# Not Everything Is Cordless Today—Case Report of Acute Intestinal Obstruction in a Neonate Due to Cord Clamping

**DOI:** 10.3389/fped.2021.810570

**Published:** 2022-01-07

**Authors:** Daniel Keese, Anne-Kathrin Schwalbach, Andrea Schmedding, Udo Rolle

**Affiliations:** Department of Pediatric Surgery and Pediatric Urology, Goethe University Frankfurt, Frankfurt, Germany

**Keywords:** intestinal obstruction, cord clamping, newborn, omphalocele, hernia to the cord

## Abstract

We report a case of a 2-day-old neonate with bilious vomiting and abdominal distension. A small bowel obstruction with ileal perforation due to a misplaced clamping of the umbilical cord was apparent before laparotomy. This complication was a sequala after clamping the cord too close to the abdominal wall in a case where there was a hernia into the cord with intestinal content. A herniation of abdominal contents due to an omphalocele minor or a hernia must be taken into consideration during the inspection of the umbilical cord before clamping.

## Introduction

Abdominal wall defects are rare congenital entities that can lead to an evisceration of intraabdominal organs. Omphalocele is an embryopathy of the midline abdominal wall. The umbilical cord inserts on the hernial sac surface. The intestine, liver, spleen, bladder or gonads can prolapse into the hernial sac (1). The hernia sac consists of the peritoneum, Wharton's sulcus and amniotic epithelium. The membranous hernia sac surrounds and protects the hernia contents. The differentiation between a small omphalocele, defined as a defect of the umbilical ring smaller than 5 cm (2), and a potential hernia into the cord is difficult. Morphologically, a hernia into the cord shows a normally configured umbilicus with intact skin and a normally configured abdominal wall (Mm recti abdominis is normal). In contrast, the abdominal wall in an omphalocele is not anatomically correct and has a missing portion of the supraumbilical fascia (3). This is because a hernia into the cord does not manifest itself until the 10th week of gestation after the supraumbilical abdominal wall has already been created. Both small omphaloceles and hernias into the cord are usually treated by a primary fascial closure with an umbilical reconstruction. There is an essential difference between an omphalocele and a hernia into the cord with respect to the associated malformations (chromosomal aberrations, heart defects, etc.).

However, mild forms of omphalocele can also present together persistent omphaloenteric duct (POD) (4). In embryological terms the POD connects the yolksac to the midgut—as a patent intestinal loop in the fetal umbilical cord—and regresses and is obliterated at the time of birth or persists as a residual Meckel's diverticulum. POD could also be affected by incorrect cord clamping.

Directly after birth, a diagnosis of herniation of the intestine due to a small omphalocele or a hernia into the cord may sometimes be overlooked when there is not a careful inspection of the umbilicus. In such circumstances, cord clamping can result in accidental injury to the intestines (5–7).

We present a case of a 2-day-old female neonate whose hernia into the umbilical cord was not recognized, and the prolapsed ileum was clamped. Due to its rarity, we want to highlight and emphasize this complication of cord clamping to prevent this type of injury at the time of birth.

Literature research was undertaken to identify similar cases with special regard to the outcome (displayed in Tables 1, 2).

**Table 1:** Reported cases from Asabe et al. (d, days; m, months; POD, persistent omphaloenteric duct).

**References**	**Age**	**Symptoms**	**Findings**	**Management of cord**	**Outcome**
Hollenberg (8)	5 d	–	Intestinal obstruction	–	Died
Hollenberg (8)	0 d	–	–	Stump	Survived
Landor et al. (9)	6 d	Abdominal distension	–	Tape	Survived
Landor et al. (9)	4 d	Bilious vomiting, abdominal distension	–	–	Died
Eckstein (10)	–	–	–	–	–
Eckstein (10)	–	–	–	–	–
Vassy and Boles (11)	3 d	Bilious vomiting, abdominal distension	–	Stump	Survived
Vassy and Boles (11)	2 d	Bilious vomiting, abdominal distension	–	Stump	Survived
Yaday and Mengi (12)	2 d	Bilious vomiting, abdominal distension	–	Ligature	Survived
Yaday and Mengi (12)	4 d	Bilious vomiting, abdominal distension	–	Ligature	Survived
Yamasato (13)	3 d	Bilious vomiting, abdominal distension	–	Stump	Survived
Chandra et al. (14)	–	–	–	Ligature	Survived
Chandra et al. (14)	4 d	–	–	Ligature	Survived
Champman-Sheath et al. (15)	3 d	Bilious vomiting, abdominal distension	–	Stump	Survived
Watanabe et al. (16)	19 d	Abdominal distension	–	Ring	Survived
Asabe et al. (7)	3 d	Bilious vomiting, abdominal distension	–	Ligature and clip	Survived

**Table 2:** Cases of literature search (d, days; m, months; POD, persistent omphaloenteric duct).

**References**	**Age**	**Symptoms**	**Findings**	**Treatment**	**Outcome**
Present case	3 d	• Bilious vomiting • Broad based umbilicus	Hernia into the cord with clamped and perforated ileum	Laparotomy with primary closure and anastomosis of perforated intestine	Survived
Kurtuluş (17)	1 d	• Unusual thickening of the umbilical cord • Refusal to drink/suck the breast	Umbilical cord hernia with clamped and perforated intestinal loop	Laparotomy with primary closure of perforated intestine	Survived
Shukla (18)	12 d	• Fecal discharge from umbilicus • Sepsis	Perforated ileum due to clamped umbilical cord hernia	Laparotomy with ileoileal anastomosis	Died (due to sepsis)
Zanatta (19)	1 d	Suspected malformed vascular vessels at the base of the umbilical cord	Resected ileal loop with partial release of the cecum and appendix	Laparotomy with end-to-end anastomosis	Survived
van Tuil et al. (4)	–	Unusual thickening of the umbilical cord/undiagnosed omphalocele	Clamped and decapitated POD	Laparotomy with resection of POD	Survived
Sandborn and Shafer (20)	4 m	Appendiceal-umbilical fecal fistula	Clamped appendix in a small unrecognized omphalocele	Right transverse laparotomy with appendectomy	Survived
Cresson and Pilling (21)	–	Umbilical fecal fistula	Small unrecognized omphalocele which had been inadvertently tied off	–	–
Williams (22)	6 d	Mass in the stump of the umbilical cord measuring 7.5 × 6 cm	Cut POD	Laparotomy with resection of POD and adhesiolysis	Survived
Jedberg (23)	2 d	• Heavy, gall-colored vomiting • No discharge of meconium • Broad based umbilicus	Tied up ileum by ligation of the cord	Laparotomy with incision paramedially to the left of the umbilicus	Died
O'Leary and Clymer (24)	–	Feca1 umbilical fistula	Ligated cord incarcerating a loop of ileum	Closure of fistula	Died
O'Leary and Clymer (24)	–	–	Ligated cord incarcerating a Meckel diverticulum	–	Survived
O'Leary and Clymer (24)	–	–	Ligated cord incarcerating a Meckel diverticulum	–	Survived
Gruss (25)	1 d	–	Thread cut through the mesentery and the transverse colon	Radical operation	Survived

## Case Report

**Figure 1:**
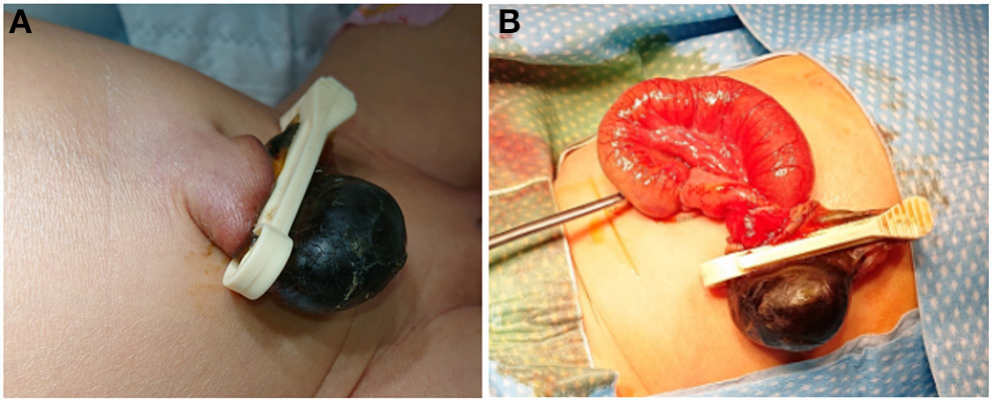
**(A)** Picture shows necrotic swelling distal to the umbilical ligation clip, which was placed ~1.5 cm from the enlarged umbilical base. **(B)** Picture taken intraoperatively showing obstruction of a small bowel loop caused by the umbilical clamp that had constricted a part of the distal ileum.

A 2-day-old girl was referred to our department of pediatric surgery from an external hospital with a 4-h history of bilious, stool-like vomiting and abdominal distension. The infant was born at term by spontaneous vaginal delivery and weighed 3,400 g. The pregnancy had been unremarkable with no history of polyhydramnios; all prenatal ultrasound scans had been normal. After birth, the newborn was in a good condition. Routine care was given to the patient, and the broad-based umbilical cord was clamped by a midwife ~2 centimeters from the umbilical base. The baby was started on breast milk feeding and had passed meconium. Twelve hours after birth, a bluish gray swelling appeared distal to the umbilical clamp, and the baby started to vomit milk and was transferred to a secondary care hospital. Twenty hours later, the patient developed bilious stool-like vomiting and abdominal distension. A nasogastric tube was passed, and the patient was transferred to our department of pediatric surgery. On the initial physical examination, the patient showed a stable general condition with a rosy skin color and normal skin turgor and capillary refill time. Heart and lung auscultation was unremarkable, and the axillary and inguinal pulses were easily palpable. Her abdomen was distended, and absent bowel sounds were noted. The umbilical cord remnant distal to the umbilical clamp appeared bluish and was necrotic and swollen, with a size of ~1.5 × 1.5 cm (Figure 1A). The anal sphincter tone was normal. A large amount of meconium was excreted during the examination. No other abnormalities were detected.

## Diagnostic Assessment and Therapeutic Intervention

Laboratory findings showed an elevated CrP level (4.83 mg/dl) and a high PCT (16.0 ng/dl). An ultrasound examination showed umbilical vessels and intestinal loops lying within the area of the umbilical clamp. IV fluids and antibiotics were initiated, and the patient was prepared for an emergency laparotomy. Intraoperatively, a loop of small bowel (ileum) that was herniated through the umbilicus was pinched off by the applied umbilical clamp and was necrotic (Figure 1B). A resection of the necrotic bowel segment and a primary end-to-end anastomosis of the ileum were performed. The infant made an uneventful recovery and was discharged to go home with planned follow up in the outpatient clinic.

## Discussion

Abdominal wall defects such as omphalocele or hernia into the cord are characterized by an abnormality of the umbilical ring (~1 in 4,000 to 1 in 10,000 births), which affects boys and girls almost equally. The sizes of omphaloceles vary considerably. Jones classified omphaloceles into defects with a diameter smaller than 2.5 cm, defects with a diameter of 2.5–5 cm and those with a diameter larger than 5 cm (2). Small omphalocele or omphalocele minor are often confused with a congenital hernia into the cord. One of the most important distinguishing features between the two is the fact that an umbilical hernia into the cord is not associated with other chromosomal anomalies in most cases and usually have a normal cord insertion with intact skin covering the umbilical ring (1). Furthermore, isolated cases are considered to have a good prognosis. On the other hand, the presence of an omphalocele is a more concerning prenatal ultrasound finding, because these cases often have abnormal karyotypes or other major anomalies. Whether there is a suspicion of a small omphalocele or a hernia into the cord, it is important to inspect the umbilicus carefully for the presence of any abdominal wall defects in the delivery room. The clinician should especially be aware of other differential diagnoses when the umbilical cord has abnormal thickening or if there is an enlargement of the umbilical cord. In addition to a careful umbilical examination, sonography is an indispensable diagnostic tool in cases of suspected abdominal wall defects and herniation of parts of the intestine. In cases of broad-based umbilicus, it is recommended that clamping or ligation of the umbilical cord should be performed at least 5 centimeters from the abdominal wall to avoid complications that have been previously reported (26). Asabe et al. reported a similar case of ileal perforation secondary to clamping of a small omphalocele in a 3-day-old girl (7). In their additional literature research, including publications from 1932 until 2007, seventeen cases were reported describing the incorrect placement of clamps or ligatures or the cutting or the excision of the cord resulting in iatrogenic damage to the bowel (Table 1). The operative treatment as well as the intraoperative findings (expect in one case) were not given. In our literature search we found additional twelve cases which also reported this complication in neonates (Table 2) (4, 8, 17–24). The first case of this injury was described by Gruss in 1927, where a hernial sac was ligated when trying to ligate the cord with a thread and which eventually cut through the mesentery and the transverse colon (8).

Altogether with our case reported here, there have been 31 reported cases of ligated hernias of the umbilical cord in the literature. A small omphalocele, a hernia into the cord, and a POD were not recognized postnatally, which resulted in the clamping or ligating herniated intestinal loops. Patients mostly had a suspected, broad-based umbilicus or presented with symptoms of abdominal distention and bilious vomiting, which are similar to the symptoms in our patient. In all cases, surgery with immediate laparotomy and exploration was the treatment of choice. Twenty patients survived (74%), and in 3 cases (7%), the outcome was not mentioned. Fatal outcomes were reported in seven cases (17%); all but one of the cases were fatal before 1963 (7, 18, 23, 24). In the newer case from 2014, the patient was already 12 days old when the diagnosis was made. The baby died 2 days postoperatively due to sepsis (18). Due to its rarity, it is essential to remember this complication and the danger and risks of serious, fatal consequences in this condition.

## Clinical Implications

The careful inspection of the umbilical cord of every newborn before clamping or ligation is essential. Obstetricians, pediatricians and nurses who clamp, manipulate, or shorten the cord should be cognizant of an abdominal wall defect with herniation of the intestine into the cord. We emphasize that in small omphaloceles or hernias into the cord, the diagnosis is not always immediately obvious. Therefore, the umbilical cord should be routinely clamped at least 5 cm from the abdominal wall to prevent any possibility of iatrogenic damage to the bowel.

## Author Contributions

DK drafted the manuscript. A-KS contributed to drafting and writing of the manuscript. AS contributed to drafting and writing. UR supervised and controlled the manuscript. All authors contributed to the article and approved the submitted version.

## Conflict of Interest

The authors declare that the research was conducted in the absence of any commercial or financial relationships that could be construed as a potential conflict of interest.

## Publisher's Note

All claims expressed in this article are solely those of the authors and do not necessarily represent those of their affiliated organizations, or those of the publisher, the editors and the reviewers. Any product that may be evaluated in this article, or claim that may be made by its manufacturer, is not guaranteed or endorsed by the publisher.

## References

[1] Raju R Satti M Lee Q Vettraino I. Congenital hernia of cord: an often misdiagnosed entity. BMJ Case Rep. (2015) 2015:bcr2015209642. 10.1136/bcr-2015-209642 25899514 PMC4420826

[2] Jones PG. Exomphalos (syn. omphalocele) A review of 45 cases. Arch Dis Child. (1963) 38:180–7. 10.1136/adc.38.198.180 13964781 PMC2018994

[3] Klein M. Congenital abdominal wall defects. In: Ashcraft KW, Holcomb GW, Murphy JP, ediotrs. Pediatric Surgery. 4th ed. Philadelphia, PA: Elsevier Saunders (2005). p. 659–69.

[4] van Tuil C Saxena AK Willital GH. Look twice before you clamp: decapitation of an omphaloenteric duct. A case report. Med Princ Pract Int J Kuwait Univ Health Sci Cent. (2006) 15:156–8. 10.1159/000090923 16484846

[5] Knight PJ Buckner D Vassy LE. Omphalocele: treatment options. Surgery. (1981) 89:332–6. 7466622

[6] Bilderback JB Rosenblatt MS. Acute intestinal obstruction caused by clamping of the intestine in the umbilical cord clamp. Ann Surg. (1946) 124:146–8. 10.1097/00000658-194607000-00014 20992175

[7] Asabe K Oka Y Kai H Shirakusa T. Iatrogenic ileal perforation: an accidental clamping of a hernia into the umbilical cord and a review of the published work. J Obstet Gynaecol Res. (2008) 34 :619–22. 10.1111/j.1447-0756.2008.00799.x 18840166

[8] Hollenberg HG. Amniotic hernia. Surgery. (1948) 23:363–8. 18906021

[9] Landor JH Armstrong JH Dickerson OB Westerfeld RA Neonatal Neonatal obstruction of bowel caused by accidental clamping of small omphalocele. report of two cases. South Med J. (1963) 56:1236–8. 10.1097/00007611-196311000-00008 14078370

[10] Eckstein HB. Exomphalos, A review of 100 cases. Br Surg J. (1963) 50:405–10. 10.1002/bjs.1800502221025855820

[11] Vassy LE Boles ET. Iatrogenic ileal atresia secondary to clamping of an occult omphalocele. J Pediatr Surg. (1975) 10:797–800. 10.1016/0022-3468(75)90387-5 1185469

[12] Yadav K Mengi Y. Iatrogenic obstruction of occult omphalocele. Indian Pediatr. (1981) 18:823–5. 7341478

[13] Yamasato M. Intestinal-umbilical fistula secondary to accidental clamping of an occult omphalocele. A case report. Ryukyu Med J. (1987) 10:112–5.

[14] Chandra S Bhatnagar V Rohatgi M. Management of omphalocele with bowel pathology–primary or iatrogenic. Indian Pediatr. (1989) 26:713–5. 2583832

[15] Chapman-Sheath P Wilcox D Mok Q Drake D. Iatrogenic ileal obstruction: a complication of umbilical cord clamping. BMJ. (1996) 313:613–4. 10.1136/bmj.313.7057.613 8806257 PMC2352044

[16] Watanabe M Komuro H Kaneko M Hori T Hirai M Urita Y. Ileal obstruction caused by usage of an umbilical ligation ring in an extremely low-birth-weight infant: a case report. (2007) 43:58−61.

[17] Kurtuluş S. Iatrogenic intestinal perforation in umbilical cord hernia. J Pediatr Surg Case Rep. (2020) 59:101500. 10.1016/j.epsc.2020.10150018840166

[18] Shukla RM. Look twice before you clamp the cord: iatrogenic ileal transection. J Obstet Gynaecol India. (2014) 64(Suppl. 1):40–1. 10.1007/s13224-013-0442-y 25404805 PMC4228040

[19] Zanatta GMC. Iatrogenic intestinal laceration secondary to clamping of unrecognized umbilical cord hernia: a case report. J Womens Health Care. (2014) 3:1000177. 10.4172/2167-0420.1000177

[20] Sandborn WD Shafer AD. Appendiceal-umbilical fistula. J Pediatr Surg. (1967) 461–3. 10.1016/S0022-3468(67)80089-7

[21] Cresson SL Pilling GP. Lesions about the umbilicus in infants and children. Pediatr Clin North Am. (1959) 1085–116. 10.1016/S0031-3955(16)30875-6

[22] Williams C. Unusual surgical lesions of the umbilicus. Ann Surg. (1946) 1108–24. 10.1097/00000658-194612000-00012 PMC1803230 17858900

[23] Jedberg H. On hernias into the umbilical cord and their treatment: Some conclusions from 9 cases. Acta Obstet Gynecol Scand. (1942) 22:283–304. 10.3109/00016344209153509

[24] O'Leary CM Clymer CE. Umbilical hernia. Am Surg J. (1941) 52:38–43. 10.1016/S0002-9610(41)90481-6

[25] Gruss J. Operativ geheilte Nabelschnurhernie mit Verletzung des Mesenteriums bei einem Neugeborenen. Ref i Zbl f Gyn. (1927) 51:244.

[26] Kirkegaard A Bjerring OS Rasmussen L. [The umbilical cord of newborn babies should be clamped at least five centimetres from the abdominal wall]. Ugeskr Laeger. (2011) 173:2270−1. 21917228

